# Brain Tumor MR Image Classification Using Convolutional Dictionary Learning With Local Constraint

**DOI:** 10.3389/fnins.2021.679847

**Published:** 2021-05-28

**Authors:** Xiaoqing Gu, Zongxuan Shen, Jing Xue, Yiqing Fan, Tongguang Ni

**Affiliations:** ^1^School of Computer Science and Artificial Intelligence, Changzhou University, Changzhou, China; ^2^Department of Nephrology, Affiliated Wuxi People’s Hospital of Nanjing Medical University, Wuxi, China; ^3^Viterbi School of Engineering, University of Southern California, Los Angeles, CA, United States

**Keywords:** brain tumor image classification, magnetic resonance imaging, dictionary learning, local constraint, convolutional neural network

## Abstract

Brain tumor image classification is an important part of medical image processing. It assists doctors to make accurate diagnosis and treatment plans. Magnetic resonance (MR) imaging is one of the main imaging tools to study brain tissue. In this article, we propose a brain tumor MR image classification method using convolutional dictionary learning with local constraint (CDLLC). Our method integrates the multi-layer dictionary learning into a convolutional neural network (CNN) structure to explore the discriminative information. Encoding a vector on a dictionary can be considered as multiple projections into new spaces, and the obtained coding vector is sparse. Meanwhile, in order to preserve the geometric structure of data and utilize the supervised information, we construct the local constraint of atoms through a supervised *k*-nearest neighbor graph, so that the discrimination of the obtained dictionary is strong. To solve the proposed problem, an efficient iterative optimization scheme is designed. In the experiment, two clinically relevant multi-class classification tasks on the Cheng and REMBRANDT datasets are designed. The evaluation results demonstrate that our method is effective for brain tumor MR image classification, and it could outperform other comparisons.

## Introduction

Brain tumors are abnormal cell aggregations that grow inside the brain tissues. Brain tumors can be divided into benign tumors and malignant tumors. Brain benign tumors can be cured by surgery, while malignant brain tumors are one of the most deadly types of cancer and can lead directly to death (Yang et al., 2018; Sun et al., 2019; Ge et al., 2020). Brain tumors can also be divided into primary tumors formed in the brain or derived from the brain nerves and metastatic brain tumors metastasized from other parts of the body to the brain. The most common primary brain tumors in adults are primary central nervous system lymphoma and gliomas, of which gliomas originate from the periglial tissue and account for more than 80% of malignant brain tumors. Different symptoms appear with different lesion areas, such as headache, vomiting, visual decline, epilepsy, and confusion. A more detailed classification divides brain tumors into four grades, the higher the grade, the more malignant the brain tumor is. According to the Global cancer statistics 2020 (Sung et al., 2021), there are about 308,000 new cases of brain cancers in 2020, accounting for about 1.6% of all new cases of cancers, and about 251,000 deaths from brain cancers, accounting for about 2.5% of all cancer deaths.

Early detection is important for effective treatment of brain tumors (Gumaei et al., 2019). With the development of medical imaging, imaging techniques play an important role in brain tumor diagnosis and treatment evaluation and can provide doctors with a clear human brain structure. These imaging techniques can provide information on the shape, size, and location of brain tumors, assisting doctors to make an accurate diagnosis and develop a treatment plan. Magnetic resonance (MR) imaging is one of the most commonly used scanning methods in neurology. MR imaging uses radiofrequency signals to excite the target tissue under the influence of a very strong magnetic field to produce an image of its interior. It has the advantages of high soft tissue contrast and zero ionizing radiation exposure. Therefore, MR imaging is more suitable for the detection of brain lesions (Zeng et al., 2018; Mittal et al., 2019; Bunevicius et al., 2020).

In recent years, artificial intelligence has attracted more and more attention due to its achievements in the field of intelligent medicine. The classification and segmentation of MR images using artificial intelligence methods has become a hot topic in the research of medical image processing (Mohan and Subashini, 2018; Anaraki et al., 2019). The application of brain tumor classification falls into two main types: classification of brain images into normal and abnormal, that is, whether the brain image contains a tumor or not; and classification within abnormal brain images, that is, differentiation between different classes of brain tumors. Classifying brain tumors into different pathological classes is more challenging than a binary classification. The challenge lies in brain tumors being permeable, their appearance is highly heterogeneous, their location is random, and the number of voxels in each subregion varies widely (Chahal et al., 2020).

Brain tumor classification includes two procedures: feature extraction and classification. In some previous studies, traditional manual extraction of features was widely used, such as intensity and texture features of brain tumor images. However, traditional feature extraction methods require the professional knowledge and experience in specific fields. Manual feature extraction will also reduce the efficiency of the system. Deep learning techniques overcome this disadvantage (Sajjad et al., 2019). Feature extraction methods based on deep learning have demonstrated successful results in real-world medical image processing applications (Deepak and Ameer, 2019). Among various classification methods, dictionary learning (DL) is a powerful tool in image processing and machine vision, making sparse coding tasks efficient and robust (Al-Shaikhli et al., 2016; Ni et al., 2020). The sparse coding can approximate high-dimensional image features into a linear combination of a few atoms from the learned dictionary (Li et al., 2018; Gu et al., 2020b). Ghasemi et al. (2020, 2021) developed fuzzy dictionaries to deal with the uncertainty in brain tumor image classification. The classic fuzzy inference is embedded into the dictionary learning process and fuzzy membership functions are used to model uncertainty and improve sparse representation. Wu et al. (2017) developed a parse representation method to exact important features and key feature index across different class images. Then, the learned feature weights and classification dictionary are used in a radiomics system for the diagnosis of brain tumors. Al-Shaikhli et al. (2014) developed a coupled dictionary learning method, which designs one dictionary of brain tumor image patches and one dictionary of image labels. The label dictionary is used to present the foreground and background multiple labels. Then, Al-Shaikhli et al. (2016) extended this work by using the information of brain topology and texture to develop a multi-class brain tumor classification method. Chen et al. (2017) proposed a kernel sparse representation method for multi-label brain tumor segmentation. This method consists of three main parts as principal component analysis—split for dictionary learning initialization, second for kernel sparse representation processing of kernel dictionary learning and kernel sparse coding, and third for making brain image segmentation using graph-cut method. Adebileje et al. (2017) applied two dictionary learning methods for classifying proton MR spectroscopy of brain gliomas tumor, i.e., one is discriminate sub-dictionary learning method, and the other is projective dictionary pair learning. These two methods were tested on many H-MRS patients signal samples selected in an Iran hospital and evaluated to be noise insensitive. Tong et al. (2019) proposed a kernel dictionary learning method that segmented MR brain tumor images after the noise removal and contrast enhancement and then extracted the nonlinear features by the learned kernel dictionary for healthy and pathologically tissues. Finally, the segmentation is done by kernel-clustering method. Vu et al. (2015) proposed a feature-oriented dictionary learning method. This method incorporated feature extraction discriminative into dictionary learning. In addition, it built discriminative class-specific dictionaries that emphasized the small intra-class differences and large inter-class differences. Finally, this method had been evaluated using brain cancer dataset Cancer Genome Atlas.

**FIGURE 1:**
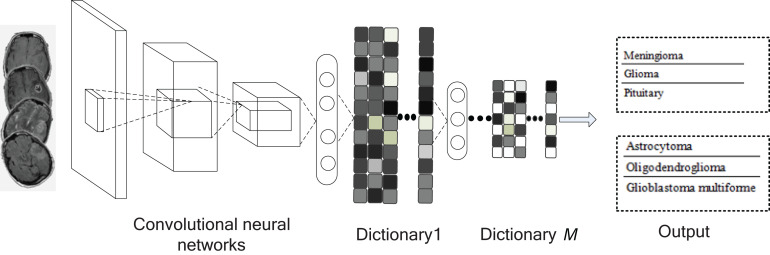
Framework of the proposed method.

Our goal in this study is to build an automatic and effective brain tumor MR image classification method to assist physicians in decision-making. In order to capture the better discriminative feature representations of brain tumor MR images, we propose convolutional dictionary learning with local constraint (CDLLC) method to seek sparse feature representation and dictionary simultaneously by using a convolutional neural network (CNN) framework. In addition, we employ the locality constraint term on codes in the last layer. The locality constraint term is used to enforce the manifold structure of the codes to preserve the locality information. Various CNN structures can be used in CDLLC; in this study, we use AlexNet (Krizhevsky et al., 2012) and softmax classifier loss in the last layer. The framework of the proposed method is shown in Figure 1. The advantages of the proposed CDLLC method are as follows: (1) CDLLC learns a multi-layer convolutional dictionary for feature representation and encoding in the nonlinear space, so that the nonlinear latent information of data is employed. (2) Encoding a vector on a multi-layer dictionary can be considered as multiple projections into new spaces. The projection is nonlinear and the obtained coding vector is sparse. Simultaneously, the resulting coding vectors of different classes can give the discriminative approximation and delete the redundant information. For example, the coding vectors in single layer dictionary learning may be nonlinear separable, and they will be transformed into linear separable in our method. (3) By considering the supervised information and graph Laplacian regularization term, the learned coding vectors are more discriminative. Simultaneously, graph Laplacian regularization preserves the locality structure information of the learned dictionary in the last layer. (4) The proposed CDLLC method is conducted on two public brain tumor datasets. The performance and usefulness of CDLLC are validated in terms of accuracy, recall, precision, F1-score, and balance loss.

The rest of the article is organized as follows: the related work is introduced in section “Backgrounds.” The proposed method is given in section “Convolutional Dictionary Learning With Local Constraint,” and experiments are reported in section “Experiments.” Finally, a conclusion is summarized in section “Conclusion.”

## Backgrounds

### Dataset

**FIGURE 2:**
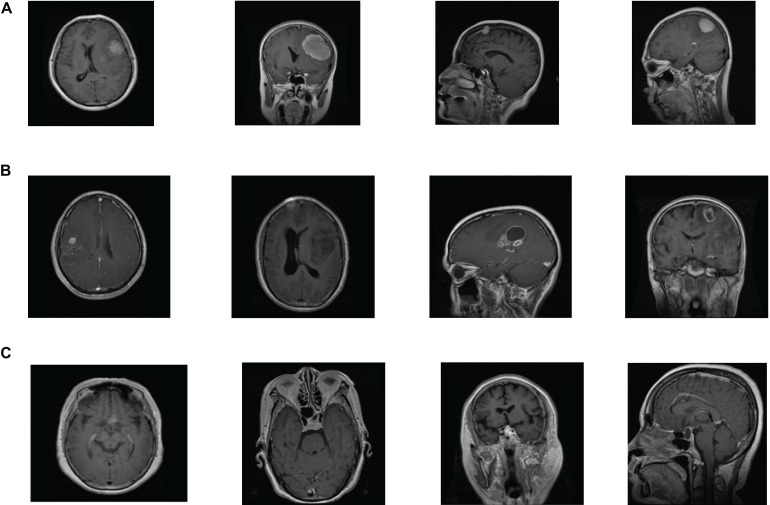
Example samples of Cheng dataset: **(A)** meningioma, **(B)** glioma, **(C)** and pituitary tumor.

**FIGURE 3:**
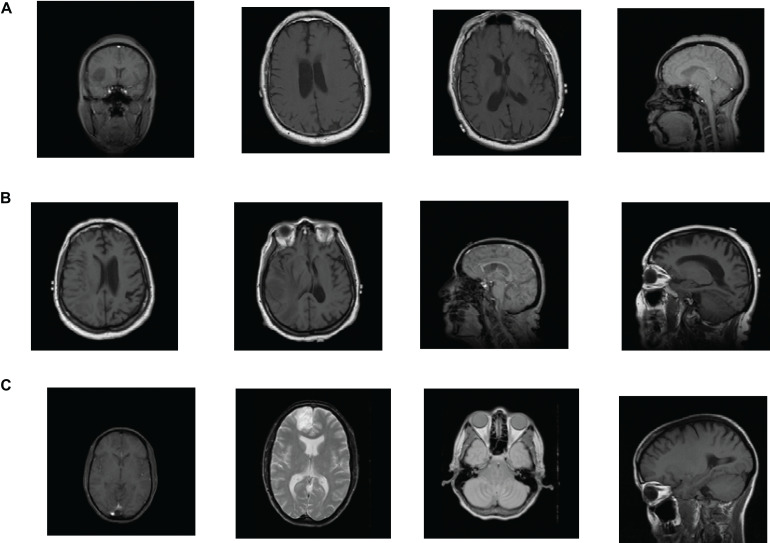
Example samples of REMBRANDT dataset: **(A)** AST, **(B)** GBM, and **(C)** OLI.

The brain tumor datasets used in this article are provided by Cheng et al. (2015, 2016) and the Repository of Molecular Brain Neoplasia Data (REMBRANDT) (Clark et al., 2013). The images provided by Cheng are 3064 T1-weighted contrast-enhanced images, containing 708 meningiomas, 1426 gliomas, and 930 pituitary tumors. All images are digitized at a resolution of 512 × 512 pixels. The REMBRANDT dataset contains 110,020 pre-surgical MR multi-sequence images from 130 brain tumor patients. The dataset contains astrocytoma (AST), oligodendroglioma (OLI), glioblastoma multiforme (GBM), and other unidentified tumor types. All images are digitized at a resolution of 256 × 256 pixels. Each image in the Cheng and REMBRANDT datasets is labeled with one type of brain tumor. The example samples of the Cheng and REMBRANDT datasets are shown in Figures 2, 3, respectively. The challenge of these two datasets lies in some factors, such as high variability in shape, size, and the similar presentation of different pathological types.

### Dictionary Learning

Let *X* = [*x*_1_,*x*_2_,…,*x*_*N*_] ∈ *R*^*d*×*N*^ be the labeled training images and *A* ∈ *R*^*K*×*N*^ be the sparse coding vector matrix. Denote the dictionary to be learned by *D* ∈ *R*^*d*×*K*^. Consider the linear representation, *DA*≈*X*. The dictionary **D** can be learned as

(1) $$\begin{array}{c} \min_{D,A}{∥\text{X-DA}∥}_{F}^{2}+\lambda \Theta \left(A\right),\\ s.t.{∥d_{i}∥}_{2}^{2}\leq 1,\forall i \end{array}$$

where the first term is reconstruction error and Θ(*A*) represents the constraints of coding vector matrix **A**, such as ℓ_0_, ℓ_1_, ℓ_2_ and Frobenius norm. The parameter λ is a positive scalar, which controls the sparsity. ${∥d_{i}∥}_{2}^{2}\leq 1$ is used to control the complexity of model. Its purpose is to prevent dictionary **D** from being arbitrarily large, as it will result in very small values for coding matrix **A**. Parameters **D** and **A** are often optimized by alternating iterations until convergence.

Equation 1 is an unsupervised learning framework. It can obtain good performance in reconstruction tasks and can also be used in some classification tasks, as it is good at mining potential patterns in the data. In order to make better use of supervised information in classification tasks, different kinds of loss functions are considered in dictionary learning. The supervised dictionary learning can be presented as

(2) $$\begin{array}{c} \min_{\theta ,D}\sum_{x\in X}L\left(l_{x},a_{x},D,\theta \right),\\ a_{x}=\arg\min{∥l_{x}-Da∥}_{2}^{2}+\lambda \Theta \left(a\right), \end{array}$$

where *l*_*x*_ is the class label of training sample **x**. Determining the suitable classification loss function *L* and its parameter **θ** are critical to classification tasks. The joint learning of parameters **D** and **θ** allows **D** to have discriminative capability with minimal classification cost. In order to obtain the optimal solution of Eq. 2, gradient descent method, back propagation method, or orthogonal matching pursuit (OMP) (Wang et al., 2012; Peng et al., 2020) can be used.

### Convolution Dictionary Learning

Convolution dictionary learning has been proposed in recent years (Papyan et al., 2018; Sulam et al., 2018; Song et al., 2019). The model is an extension of traditional dictionary learning. Its aim is to capture the deep structure of data and increase the discriminability of features. Convolution dictionary learning follows the architecture of CNN and is used in a hierarchical way. The convolution of the filter in CNN corresponds to the sparse coding step in multi-layer convolution dictionary. Let ${\left\{D_{m}\right\}}_{m=1}^{M}$ be *M*-layer convolution dictionary, where *D*_*m*_ ∈ *R*^*d*×*K*_*m*_^ is the dictionary in the *m*-th layer dictionary and *K*_*m*_ is the size of *D*_*m*_. The convolutional representation of **X** can be presented as *X*≈*D*_1_*D*_2_…*D*_*M*_*A*_*M*_. In detail, with the decomposition constraint *D*_*m*−1_ = *D*_*m*_*A*_*m*_, this process can be described as

(3) $$\begin{array}{c} X\approx D_{1}A_{1},\\ X\approx D_{1}D_{2}A_{2},\\ X\approx D_{1}D_{2}D_{3}A_{3},\\ \vdots \\ X\approx D_{1}D_{2}\ldots D_{M}A_{M}. \end{array}$$

where *A*_*m*_ ∈ *R*^*K*_*m*_×*N*^ is the coding vector matrix in the *m*-th layer.

## Convolutional Dictionary Learning With Local Constraint

### Objective Function

Consider multi-layer dictionary architecture with *M* layers, the coding *A*_*m*_ in the *m*-th layer can be written as

(4) $$A_{m}\approx \phi \left(D_{m+1}A_{m+1}\right),$$

where ϕ is a nonlinear function, such as rectified linear unit (ReLU), Sigmoid, and TanHyperbolic activation function. In this case, coding using a dictionary is used as a projection into another feature space, and the coding vector is a new input for the next layer. In order to preserve the essential structure information of data, it is essential to reconstruct the original sample in the last layer. We consider the following equation:

(5) $$\begin{array}{c} J_{1}\left(D_{1},D_{2},\ldots ,D_{M},A_{M}\right)={∥{\text{X-D}}_{1}\phi \left(D_{2}\phi \left(\ldots \phi \left(D_{M}A_{M}\right)\right)\right)∥}_{F}^{2}\\ \;+\lambda \Theta \left(A_{M}\right), \end{array}$$

where λ is the regularization parameter.

Following Cai et al. (2013) and Ding and Fu (2018), we use the rank operator for Θ(*A*_*M*_). In this case, *A*_*M*_ can be approximated as *A*_*M*_≈*SH*, and the Θ(*A*_*M*_) term can be written as

(6) $${∥A_{M}-SH∥}_{F}^{2},$$

where *S* ∈ *R*^*K*_*M*_×*C*^ and *H* ∈ *R*^*C*×*N*^. *C* is the class number of data samples.

To improve the classification performance, local information takes an important part in dictionary learning (Peng et al., 2020). Since the atoms of dictionary is more robust and stable than original samples, we use a graph Laplacian regularization term of atoms to trace the manifold structure of data. We build a supervised *k*-nearest neighbor graph **W** of atoms on dictionary *D*_*M*_ in the last layer. The element *w*_*i*,*j*_ in graph **W** is denoted as follows:

(7) $$w_{i,j}=\left\{ \begin{array}{cc} \text{exp}\left(-\frac{{∥d_{M,i}-d_{M,j}∥}^{2}}{\sigma }\right), & \text{if}\;d_{M,i}\in KNN\left(d_{M,j}\right)\\ & \text{and}\;l_{i}=l_{j}\\ 0, & \text{else} \end{array}\right.,$$

where σ is an adjustable parameter. Different from the unsupervised graph Laplacian (Peng et al., 2020), we embed the supervised information in graph **W**, such that similar codes are enforced and more discriminative information can be learned.

Then, we construct the graph Laplacian regularization term in the last layer as follows:

(8) $$J_{2}\left(A_{M}\right)=\sum_{i=1}^{K_{M}}\sum_{j=1}^{K_{M}}w_{i,j}{∥a_{M,i}-a_{M,j}∥}_{2}^{2}=Tr\left(A_{M}^{T}LA_{M}\right),$$

where the Laplacian matrix *L* = *diag*(*w*_1_,*w*_2_,…,*w*_*K*_*M*__)−*W*, and $w_{i}=\sum_{j=1}^{K_{M}}w_{i,j}$.

To further learn a discriminative dictionary by exploiting supervised information, we use the softmax classifier loss in the last layer, which is commonly used in CNN structure

(9) $$J_{3}\left(A_{M},\theta \right)=-\frac{1}{M}\sum_{i=1}^{M}\sum_{c=1}^{C}p_{c,i}log\frac{e^{\theta_{c}^{T}a_{M,i}}}{\sum_{u=1}^{C}e^{\theta_{u}^{T}a_{M,i}}},$$

where *p*_*c*,*i*_ is the label probability of *a*_*M*,*i*_ and is assigned to class *c*. θ = [θ_1_,θ_2_,…,θ_*C*_] is the adjustable parameter in softmax classifier.

For brain tumor image classification, we combine the three functions *J*_1_, *J*_2_, and *J*_3_ together, jointly optimizing convolutional dictionary learning and classifier. Therefore, we have the objective function of the CDLLC model

(10) $$\begin{array}{c} \arg\min J_{1}\left(D_{1},D_{2},\ldots ,D_{M},A_{M}\right)+J_{2}\left(A_{M}\right)+J_{3}\left(A_{M},\theta \right),\\ s.t.{∥d_{i}∥}_{2}^{2}\leq 1,\forall i \end{array}$$

To show all terms clearly, we expand the expression as follows:

(11) $$\begin{array}{c} \left[D_{1},D_{2},\ldots ,D_{M},A_{M},\theta \right]\\ =\arg\min{∥{\text{X-D}}_{1}\phi \left(D_{2}\phi \left(\ldots \phi \left(D_{M}A_{M}\right)\right)\right)∥}_{F}^{2}+\lambda_{1}{∥A_{M}-SH∥}_{F}^{2}\\ \;+\lambda_{2}Tr\left(A_{M}^{T}LA_{M}\right)-\frac{\lambda_{3}}{M}\sum_{i=1}^{M}\sum_{c=1}^{C}p_{c,i}\text{log}\frac{e^{\theta_{c}^{T}a_{M,i}}}{\sum_{u=1}^{C}e^{\theta_{u}^{T}a_{M,i}}},\\ s.t.{∥d_{i}∥}_{2}^{2}\leq 1,\forall i \end{array}$$

This jointly optimizing convolutional dictionary learning and classifier has benefits. During the procedure of optimizing, the convolutional dictionary learning gradually enhances the classification performance of the classifier; meanwhile, the learned classifier also improves the numerical stability and discriminative ability of dictionary coding.

### Optimization of CDLLC

The optimization of Eq. 11 is not convex, and we solve dictionaries {*D*_1_,*D*_2_,…,*D*_*M*_}, coding matrix **A** and classifier parameter θ by an alternative optimization approach. In each step of iteration, we compute a certain parameter and fix the other parameters.

When fixing **A** and θ, we update dictionaries {*D*_1_,*D*_2_,…,*D*_*M*_}. We use the chain rule to compute *D*_*m*_(1≤*m*≤*M*) in each layer

(12) $$\begin{array}{c} \frac{\partial J}{\partial D_{m}}=\frac{\partial J}{\partial \left(D_{m}A_{m}\right)}A_{m}^{T}\\ =\left[\frac{\partial J}{\partial \phi \left(D_{m}A_{m}\right)}⊙\phi^{\prime }\left(D_{m}A_{m}\right)\right]A_{m}^{T}\\ =\left[\frac{\partial J}{\partial A_{m-1}}⊙\phi^{\prime }\left(D_{m}A_{m}\right)\right]A_{m}^{T}, \end{array}$$

where ⊙ denotes the element-wise multiplication. Specifically, we can obtain $\frac{\partial J}{\partial D_{1}}=2\left(D_{1}A_{1}-X\right)A_{1}^{T}$. After dictionary *D*_*M*_ is obtained, we use Eq. 7 to construct the graph Laplacian regularization.

When fixing {*D*_1_,*D*_2_,…,*D*_*M*_} and θ, we update coding matrix *A*_*m*_(1 < *m*≤*M*−1) in each layer as

(13) $$\begin{array}{c} \frac{\partial J}{\partial A_{m}}={\left(D_{m}\right)}^{T}\frac{\partial J}{\partial \left(D_{m}A_{m}\right)}\\ ={\left(D_{m}\right)}^{T}\left[\frac{\partial J}{\partial \phi \left(D_{m}A_{m}\right)}⊙\phi^{\prime }\left(D_{m}A_{m}\right)\right]\\ ={\left(D_{m}\right)}^{T}\left[\frac{\partial J}{\partial A_{m-1}}⊙\phi^{\prime }\left(D_{m}A_{m}\right)\right]. \end{array}$$

Specifically, we can obtain *A*_1_ as

(14) $$\frac{\partial J}{\partial A_{1}}=2D_{1}^{T}\left(D_{1}A_{1}-X\right).$$

The partial derivatives of the *J* regarding the *A*_*M*_ is given as

(15) $$\begin{array}{c} \frac{\partial J}{\partial A_{M}}=2{\left(D_{M}\right)}^{T}\left[\frac{\partial J}{\partial A_{M-1}}⊙\phi^{\prime }\left(D_{M}A_{M}\right)\right]+2\lambda_{1}\left(A_{M}-SH\right)\\ +2\lambda_{2}L_{M}A_{M}-\frac{\lambda_{3}}{M}\sum_{c=1}^{C}\left(\theta_{c}-\frac{\sum_{u=1}^{C}\theta_{u}e^{\theta_{u}^{T}A_{M,i}}}{\sum_{u=1}^{C}e^{\theta_{u}^{T}A_{M,i}}}\right). \end{array}$$

Considering Eq. 6, we need to update matrixes **S** and **H** in turn. The optimal **S** can be computed as

(16) $$\frac{\partial J}{\partial S}=A_{M}H^{T}-SHH^{T}.$$

Then, we can update **S** as

(17) $$S=A_{M}H^{T}{\left(HH^{T}\right)}^{†},$$

where † is the Moore–Penrose pseudoinverse.

Similarly, the optimal **H** can be computed as

(18) $$\frac{\partial J}{\partial H}=S^{T}SH-S^{T}A_{M}.$$

Then, we can update **S** as

(19) $$H={\left(SS^{T}\right)}^{†}S^{T}A_{M}.$$

In this study, we use the softmax classifier for brain tumor image classification. The optimal classifier parameter **θ** can be computed as

(20) $$\frac{\partial J}{\partial \theta_{c}}=-\frac{1}{M}\sum_{i=1}^{M}\sum_{c=1}^{C}l_{c,i}\left(1-\frac{e^{\theta_{c}^{T}a_{M,i}}}{\sum_{u=1}^{C}e^{\theta_{u}^{T}a_{M,i}}}\right)\alpha_{M,i}^{T}.$$

### Testing Brain Tumor MR Images

Let **x** be the feature descriptor of a test image, based on the learned optimal **S**, **H**, θ, and {*D*_1_,*D*_2_,…,*D*_*M*_}, we can compute the dictionary encoding of **x** by the following formulation:

(21) $$\min_{a_{M}}{∥{\text{x-D}}_{1}\phi \left(D_{2}\phi \left(\ldots \phi \left(D_{M}a_{M}\right)\right)\right)∥}_{F}^{2}+\lambda_{1}{∥a_{M}-SH∥}_{F}^{2}.$$

Finally, the softmax classifier is used for classification, and the label probability of **x** assigned to class *c* can be computed as

(22) $$p_{c}=\frac{e^{\theta_{u}^{T}a_{M}}}{\sum_{u=1}^{C}e^{\theta_{u}^{T}a_{M}}}.$$

## Experiments

### Experimental Settings

In our experiment, we randomly select 1000 images in the REMBRANDT dataset and the whole Cheng dataset for simulation. Brain MR images are resized to 227 × 227 sizes to use for AlexNet. All convolution layers in CDLLC employ filters of size 3 × 3. Stochastic gradient descent (SGD) is used as an optimizer and TensorFlow is implemented in model training. ReLU is used as the activation function. The initialization of dictionary is performed from **D**_1_ and **A**_1._ We run K-SVD algorithm on each class of data and integrate subclass dictionary into the dictionary **D**_1_. Then, we obtain the **D***_*m*_* and **A***_*m*_* based on the learned dictionary and coding from the previous layer. The parameters λ_1_, λ_2_, and λ_3_ in CDLLC are selected in the search grid {0.001, 0.01, 0.1, 1, 10}. We use the fivefold cross-validation to complete with valid and comparable results. The 70% of the training fold are used for training and 30% are used as validation. We perform experiments for 10 times and then record their average values. We summarize the performance of all comparative methods in terms of accuracy, F1-score, precision, recall, and balance loss (Gu et al., 2018, 2020a).

There are many categories of methods used for brain tumor MR image classification. We compare several traditional machine learning and deep learning methods in the experiments. The traditional machine learning methods include the support vector machine with RBF kernel (SVM-RBF) (Nandpuru et al., 2014), the dictionary learning method label consistent K-SVD (called LC-KSVD1 in the experiment) (Jiang et al., 2013), and the neural network fuzzy inference system (ANFIS) (Thirumurugan and Shanthakumar, 2016). The deep learning classification methods include CNN (Krizhevsky et al., 2012) and CapsNet (Afshar et al., 2018). For these traditional classification methods, feature selection is a key step, where first- and second-order statistical texture features are widely used in brain tumor image classification. In our experiment, we follow (Fujima et al., 2019; Zhang et al., 2019) and use the statistical texture features: mean, variance, standard deviation, skewness, kurtosis, contrast, energy, entropy, correlation, and homogeneity. In addition, to compare with LC-KSVD using deep features, we exploit features from CNN architecture AlexNet and apply these features into LC-KSVD (called LC-KSVD2 in the experiment). We conduct the experiments on a computer with Intel Xeon Processor E5-2620 v4 and 64 GB RAM. All methods are implemented on Python 2.7, using Keras library and Tensor Flow.

### Experimental Results on Cheng Dataset

In this subsection, we observe the classification performance of CDLLC on the Cheng dataset. The images of the Cheng dataset used in our experiment contain three types of brain tumors: meningioma, glioma, and pituitary. First, we show the confusion matrix for three classes obtained by CDLLC in Table 1. The confusion matrix provides the valuable information about the predicted labels. From Table 1, we can see that the pituitary tumor is classified with the highest accuracy, glioma tumors are classified with the second accuracy, and meningioma tumors are classified with the lowest accuracy. Generally, the overall classification accuracy is satisfactory.

**TABLE 1:** Confusion matrix of CDLLC on the Cheng dataset.

	Meningioma	Glioma	Pituitary
Meningioma	0.8875	0.0782	0.0343
Glioma	0.0444	0.9487	0.0069
Pituitary	0.0093	0.0070	0.9837

**FIGURE 4:**
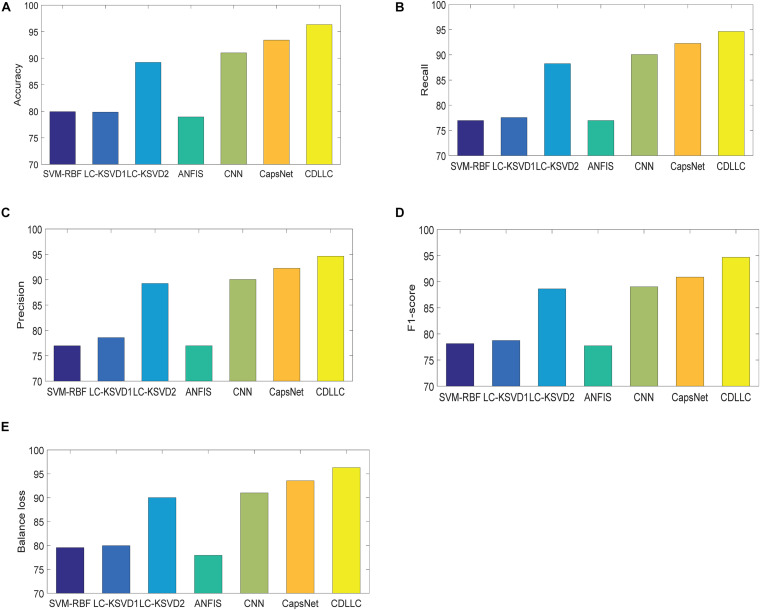
Performance of CDLLC on the Cheng dataset: **(A)** accuracy, **(B)** recall, **(C)** precision, **(D)** F1-score, and **(E)** balance loss.

Second, we use accuracy, F1-score, precision, recall, and balance loss as the evaluation indexes. The performance of these five indexes of CDLLC in five folds is shown in Table 2 in detail. Table 2 shows that CDLLC obtains high average values and small standard deviation on accuracy, F1-score, precision, recall, and balance loss. Then, we compare our method with RBF-SVM, LC-KSVD1, LC-KSVD2, ANFIS, CNN, and CapsNet. The average experimental results on the Cheng dataset are shown in Figure 4. It can be observed from these results that (1) CDLLC achieves the best results in comparison with other methods. This indicates that more discriminative information can be exploited by the proposed method. It suggests that the multi-layer dictionary learning, which addresses both feature representation and encoding in the nonlinear space, can exploit discriminative features from deep learning structure. In addition, graph Laplacian regularization can preserve the locality structure information of sparse codes, which can largely improve the model discriminative ability. (2) Among all methods, deep leaning methods gain better classification performance than traditional machine learning (SVM-RBF, LC-KSVD1, and ANFIS) with statistical texture features. Using the deep features, the classification performance of LC-KSVD2 is obviously improved than LC-KSVD1. It indicates that deep features are more adapted to brain tumor image classification.

**TABLE 2:** Classification performance of CDLLC on the Cheng dataset.

		Accuracy	Recall	Precision	F1-score	Balance loss
Fold-1	Training	96.94	94.97	95.17	94.95	96.56
	Test	96.36	94.59	94.78	94.70	96.03
Fold-2	Training	96.76	95.15	94.78	95.25	96.44
	Test	96.29	94.71	94.42	94.63	96.08
Fold-3	Training	96.77	95.02	95.21	95.30	96.41
	Test	96.32	94.62	94.51	94.65	96.11
Fold-4	Training	96.83	94.89	94.99	95.10	96.47
	Test	96.35	94.67	94.60	94.75	96.06
Fold-5	Training	97.12	95.09	95.28	95.17	96.96
	Test	96.39	94.64	94.61	94.70	96.22

### Experimental Results on REMBRANDT Dataset

In this subsection, we observe the classification performance of CDLLC on the REMBRANDT dataset. The images of the REMBRANDT dataset used in our experiment contain three types of brain tumors: AST, OLI, and GBM.

**FIGURE 5:**
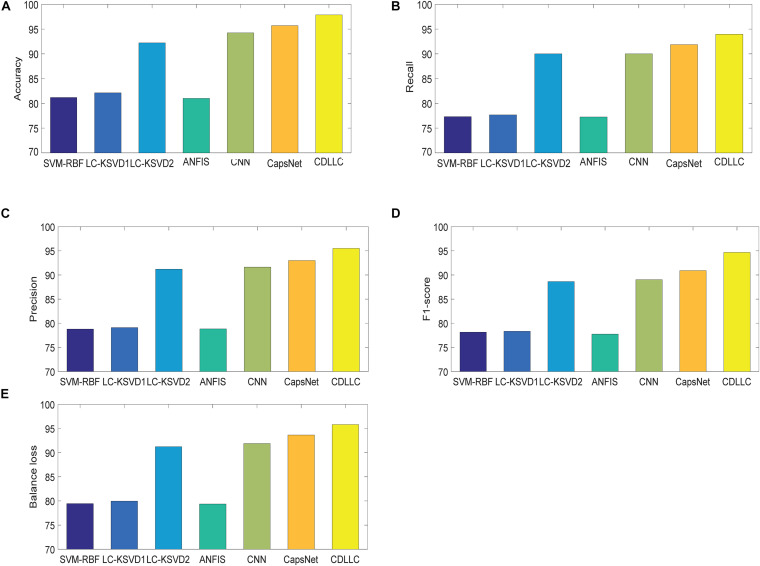
Performance of CDLLC on the REMBRANDT dataset: **(A)** accuracy, **(B)** recall, **(C)** precision, **(D)** F1-score, and **(E)** balance loss.

We first show the confusion matrix for three classes obtained by CDLLC in Table 3. From Table 3, we can see that the classification performance of CDLLC for three types of brain tumor images is comparable. The classification rates of AST, OLI, and GBM are 0.9686, 0.9127, and 0.9309, respectively. Second, we summarize the performance of CDLLC in terms of accuracy, F1-score, precision, recall, and balance loss in Table 4. From the fivefold results in Table 4, we can see that CDLLC gains the satisfactory results on the REMBRANDT dataset. Our multi-layer dictionary structure is not only convolutional but also sparse, and all parameters are updated within joint optimization learning. Then, we compare our method with RBF-SVM, LC-KSVD, ANFIS, CNN, and CapsNet. The average experimental results on the REMBRANDT dataset are shown in Figure 5. Similar to the results in Figure 4, CDLLC gains the best performance in five evaluation indexes. LC-KSVD1 is as a baseline method of our method. Whether using statistical texture features or deep features, the performance of LC-KSVD1 is much lower than that of the proposed CDLLC. The reason is that LC-KSVD1 learns the dictionary in the original space and such dictionary cannot well exploit the discriminative information.

**TABLE 3:** Confusion matrix of CDLLC on the REMBRANDT dataset.

	AST	OLI	GBM
AST	0.9686	0.0100	0.0214
OLI	0.0621	0.9127	0.0252
GBM	0.0583	0.0108	0.9309

**TABLE 4:** Classification performance of CDLLC on the REMBRANDT dataset.

		Accuracy	Recall	Precision	F1-score	Balance loss
Fold-1	Training	97.74	93.89	95.42	94.25	95.86
	Test	97.55	93.67	95.28	94.10	95.39
Fold-2	Training	97.80	93.92	95.53	94.34	95.91
	Test	97.74	93.90	95.38	94.21	95.39
Fold-3	Training	97.87	93.96	95.52	94.35	95.82
	Test	97.74	93.89	95.39	94.22	95.56
Fold-4	Training	97.85	93.97	95.58	94.47	95.99
	Test	97.80	93.92	95.52	94.34	95.71
Fold-5	Training	97.74	93.90	95.48	94.26	95.79
	Test	97.37	93.66	95.12	94.05	95.42

### Parameter Analysis

**FIGURE 6:**
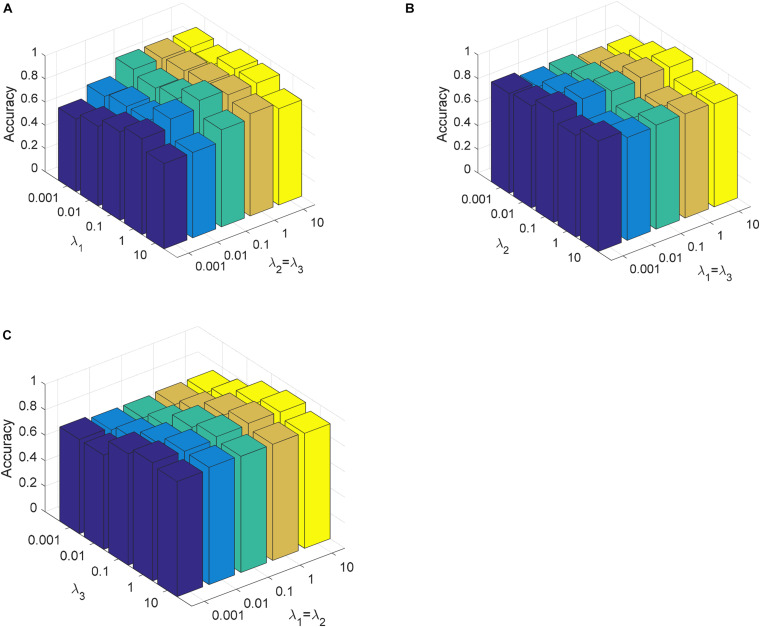
Parameter sensitivity of CDLLC on the Cheng dataset: **(A)** λ_1_ and λ_2_ (λ_3_), **(B)** λ_2_ and λ_1_ (λ_3_), and **(C)** λ_3_ and λ_1_ (λ_2_).

**FIGURE 7:**
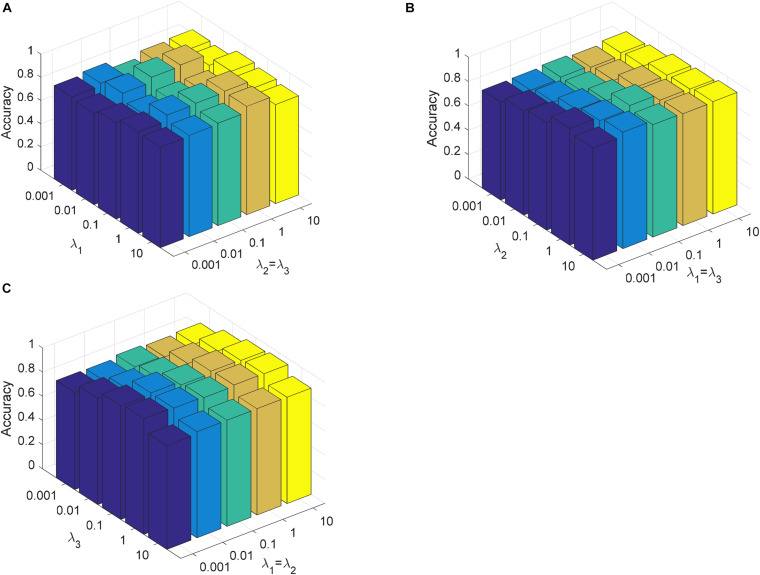
Parameter sensitivity of CDLLC on the REMBRANDT dataset: **(A)** λ_1_ and λ_2_ (λ_3_), **(B)** λ_2_ and λ_1_ (λ_3_), and **(C)** λ_3_ and λ_1_ (λ_2_).

In this section, we analyze the parameter sensitivity of CDLLC on the Cheng and REMBRANDT datasets. First, we discuss the parameters λ_1_, λ_2_, and λ_3_ in CDLLC. These three parameters are selected in the search grid {0.001, 0.01,…, 10}. We set λ_1_ = λ_2_ and visualize the change of classification accuracy of CDLLC with different values of λ_2_ and λ_3_. Similarly, we set λ_2_ = λ_3_ (λ_1_ = λ_3_) and visualize the change of classification accuracy of CDLLC with different values of λ_1_ and λ_3_ (λ_1_ and λ_2_). The results of classification accuracy are shown in Figures 6, 7. We can see that different values of the parameters λ_1_, λ_2_, and λ_3_ have a significant impact on the classification accuracy of CDLLC. It indicates that the grid search strategy is appropriate for λ_1_, λ_2_, and λ_3_.

**FIGURE 8:**
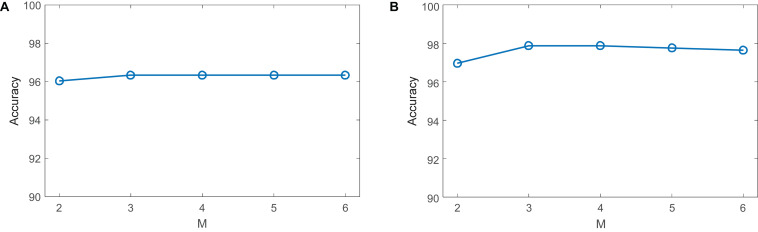
Parameter sensitivity *M* of CDLLC on **(A)** the Cheng dataset and **(B)** the REMBRANDT dataset.

Next, we discuss the number of layers *M* in CDLLC on the Cheng and REMBRANDT datasets. We set *M* in grid {2, 3,…, 6} to evaluate its effect on classification accuracy. The classification result is shown in Figure 8. We see that the classification accuracy of CDLLC improves when *M* increases from 1 to 3. When *M* is greater than 3, the classification accuracy of CDLLC is reliable on the Cheng and REMBRANDT datasets.

## Conclusion

In this study, we propose CDLLC method for brain tumor MR image classification. The CNN structure is utilized to seek sparse representation in the nonlinear space, so that the resulting coding vectors of different classes can give the discriminative approximation. Meanwhile, the proposed method CDLLC uses the locality constraint of atoms to preserve the manifold structure of the codes. Different from the traditional dictionary learning that uses manual feature extraction, CDLLC extracts the useful CNN features automatically in the architecture of deep learning. Classification of types of meningiomas, gliomas, and pituitary tumors on the Cheng dataset and types of AST, OLI, and GBM on the REMBRANDT dataset is carried out with high performance in accuracy, recall, precision, F1-score, and balance loss. The shortcoming of CDLLC is that the selection of parameters becomes complicated as the number of layers increases. In our next work, we will try to design a more reasonable program to select these parameters. Besides, we will compare various network architectures on CDLLC, such as VGG and GoogLeNet. Also, we will adjust our method so that it could be applied to other medical MR images.

## Data Availability Statement

Publicly available datasets were analyzed in this study. This data can be found here: The Cheng dataset analyzed for this study can be found at: https://figshare.com/articles/dataset/brain_tumor_dataset/1512427. The REMBRANDT dataset analyzed for this study can be found at: https://wiki.cancerimagingarchive.net/display/Public/REMBRANDT.

## Author Contributions

TN and XG conceived and developed the theoretical framework of the manuscript. All authors carried out experiment and data process, and drafted the manuscript.

## Conflict of Interest

The authors declare that the research was conducted in the absence of any commercial or financial relationships that could be construed as a potential conflict of interest.
